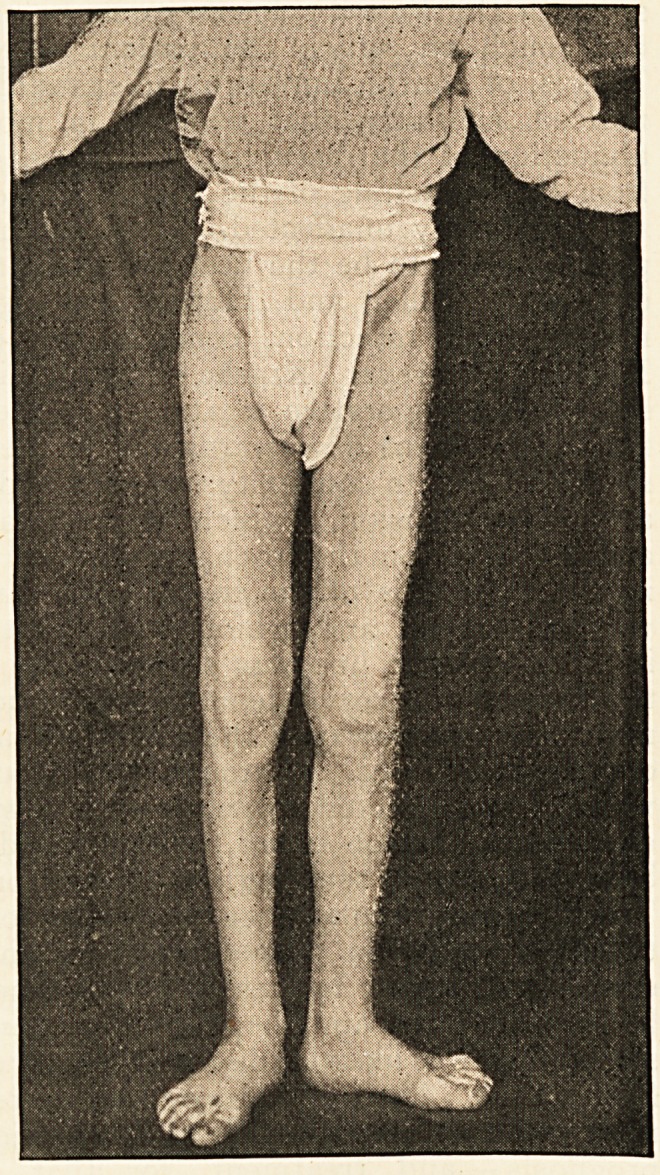# Surgery

**Published:** 1896-12

**Authors:** James Swain


### SURGERY.

**Diseases of the hip-joint** still require elucidation, but Professor Ogston[3] has advanced them a stage by the publication of several cases of "coxa vara"—a name first suggested by Hofmeister[4] in 1894 for this disease, which was described by Müller[5] in 1889.

From time to time one comes across cases which have a great resemblance to the ordinary morbus coxæ of childhood or young adult life, but which are sufficiently atypical for us to enter them in our case-books with a note of interrogation after them. I find that during the past four years I have seen some half-dozen of such cases, and which I have been disposed to regard—in the absence of other explanation—as of a "rheumatic" nature. The first case that impressed me was in a boy of about twelve years of age, who complained of pain in the right hip and knee of a few weeks' duration. He walked with the usual limp, and the limb was wasted and everted, but there was an absence of that alteration of the gluteal and inguinal folds, of lordosis, and of abduction associated with eversion which characterise a typical case of morbus coxæ. The child was put to bed, and improved; but it was thought desirable to order a Thomas's splint at the end of a week or ten days. He could not, however, be kept quiet, and in the course of a few weeks was brought back apparently well. The condition, however, recurred, and such recurrence—or more often exacerbation and remission without complete intermission—has been a

[2] *Méd. mod.*, Feb. 22, 1896. [3] *Practitioner*, 1896, lvi. 347.

[4] *Beitr. z. klin. Chir.*, 1894, xii. 245; 1895, xiii. 289. [5] *Ibid.*, 1888–9, iv. 137.

marked feature of those cases of which I have had experience. Coxa vara appears to be due to a downward curvature of the cervix femoris, of rachitic origin—as shown by the existence of "beaded ribs" and other signs of rickets. Ogston[1] says he has never found the enlargement of the ribs absent, and therefore rightly regards this objective evidence as of great diagnostic importance in these cases. The symptoms usually present in coxa vara are suggested by the case referred to above, but reference may be made to others which Prof. Ogston points out on the authority of Hofmeister, corroborated by his own observations. The disease, which may affect one or both hip-joints, comes on gradually, and usually occurs between the ages of thirteen and eighteen. The pain, which also affects the knee, is increased on standing and walking. The curving of the neck of the femur causes a shortening of the limb above the trochanter, varying from one to three inches. This is best demonstrated by means of the well-known Bryant's triangle. Eversion of the limb is present in most cases; but Hofmeister lays stress on the absence of abduction, which is present in those cases of hip-disease which are likely to be confounded with coxa vara. (A well-marked example of this disease is shown in the accompanying picture,[2] taken from a patient who was in the Aberdeen Royal Infirmary last year.) The power of abduction is generally diminished or abolished; "and the range of rotation at the

[1] *Loc. cit.*

[2] I am indebted to the courtesy of the Editor of the *Practitioner* for the loan of the block.

hip-joint, without being lessened, is simply displaced outwards." Signs of rickets, such as enlarged bones, and sometimes genu valgum and talipes valgus, are present. The persistence of the disease has been referred to, and when at the end of a few months to as many years the pain disappears, the deformity still remains. Professor Ogston also states that in his experience these cases do not exhibit that marked flexion of the hip-joint which is so common a feature in cases of morbus coxæ; so that in cases of coxa vara we find that when the knee of the *sound* side is pressed against the thorax, the popliteal space of the *diseased* side can easily be pressed into contact with the couch on which the patient is lying. (Thomas's test.) This is, of course, impossible in cases of morbus coxæ. I have noticed, similarly, that whereas in morbus coxæ flexion and abduction of the diseased limb cause great pain (presumably from the stretching of the round ligament), cases of coxa vara are practically unaffected. Rest, extension, and tonics (phosphorus) seem to be the best means of treating this interesting disease. Müller and Hoffa,[1] although they obtained good results from resection, consider the operation unjustifiable. Recently, however, Kraske[2] has published a case which he subjected to operative treatment in the belief that the cervix femoris is "bent downwards and at the same time backwards, so as to have its convexity upwards and forwards."[3] He made a two-inch incision, "which began at a point a little upwards and inwards from the trochanter tip and was carried downwards in the long axis of the limb at the outer edge of the tensor vaginæ femoris, and which was deepened until the anterior intertrochanteric line was reached, the periosteum in front of the base of the cervix femoris was divided and pushed off the neck upwards towards the caput for about an inch, without opening the joint. Through this wound a wedge of bone, whose base, directed forwards and upwards, was about three-quarters of an inch broad, was chiselled out of the bone at the base of the neck, severing the cervix from the rest of the femur. The deformity was at once, according to Kraske, overcome and the limb placed in proper position; and the case, treated by distraction, was so far healed at the time of his report that the bone had united and the joint was movable, and the opposite side, which was likewise diseased, was about to be operated upon forthwith."[4] The importance of a due recognition and differentiation of this new affection of the hip-joint is manifest, for the prognosis—and treatment, for that matter—is essentially different from that of morbus coxæ; and its diagnosis is not difficult now that its objective signs have been made known. Karl Baur has recently issued a small monograph[5] on this subject.

[1] Quoted by Prof. Ogston. [2] *Centralbl. f. Chir.*, 1896, xxiii. 121.
[3] *Practitioner*, 1896, lvi. 358. [4] *Ibid.*
[5] *Ein Beitrag zur Casuistik der Coxa vara.* 1895.

The **surgery of the vermiform appendix** has frequently been referred to in this *Journal*, but it is only by the patient accumulation of the evidence of experience, and reviewing our position from time to time, that we can proceed with a prospect of obtaining the best results. Already there are indications of the adoption of a compromise between the comparatively late operation of English surgeons and the immediate resort to operative interference of the Americans. Most surgeons are agreed on the necessity of operating under certain circumstances, such as the presence of pus, in cases of relapsing appendicitis, and so forth; but many points remain unsettled, and one that has recently been exercising the minds of some of the American surgeons is whether the appendix should be removed in every case of appendicular abscess or not. In a paper[1] read before the Bath and Bristol Branch of the British Medical Association in 1894, I stated that the rule should be to take the appendix away "if it can be removed without increasing the danger to the patient," but that "it would be far wiser not to attempt the removal of the appendix at all, than to run any great risk of opening the general peritoneal cavity in the separation of adhesions." Since then many heated discussions have gathered round this point, some supporting the view here mentioned, and others urging the removal of the appendix under all circumstances. One writer says[2]: "It is my practice to remove the appendix in all cases. Certain it is that by the proper disposition of gauze and careful attention to technic, an appendix which is deeply embedded in a wall of lymph, whether it makes a portion of the abscess-cavity or not, can be removed, and the dangers which attend its removal are far less than those which occur when it is allowed to remain. A practice which I believe is a frequent one is simply to evacuate the collection, there being no attempt made to remove the appendix if it be not plainly visible. This I consider, with all due deference to the surgeons who practise it, incomplete surgery." On the other hand, we hear[3] that "In many cases of circumscribed abscess, and especially in those in which the appendix is bound down by adhesions in the depth of the wound, the surgeon should be content with evacuation, irrigation, drainage, and packing with iodoform gauze. Persistent search for the appendix and attempts at its removal in these cases are attended with such danger of opening the general peritoneal cavity that they are not to be recommended." Dr. William White has taken much pains to collect evidence which, in the sequel, will, I think, be shown to settle this thorny question; and the paper[4] which he read at the College of Physicians of Philadelphia brought out the opinions of many of

[1] *Bristol M.-Chir. J.*, 1894, xii. 9. [2] *Med. News*, 1895, lxvii. 677.
[3] *An American Text-Book of Surgery.* Edited by W. W. Keen and J. W. White. 2nd Edition, 1895, p. 746.
[4] *Ann. Surg.*, 1896, xxiii. 759.

the leading American surgeons on this point. Most operating surgeons will agree with Dr. White that there is ample proof that it is absolutely impossible to remove the appendix "in all cases" with less danger than would ensue if it were left, and the literature of the subject is full of cases where no harm has followed when the appendix has been left behind. Fowler has reported[1] seventeen such cases which recovered, and of these two only had a recurrence within the next two years, and some of them had gone for three or four years without any return of the symptoms. Richardson, too, reported[2] forty cases in which he did not remove the appendix, recovery taking place in nearly every instance, all fæcal fistulæ ultimately healing, and subsequent trouble occurring only in two cases. Speaking of the 181 cases which he reported, Richardson says[3]: "Most surgeons believe that in cases of localized peritonitis no attempt should be made to separate the adhesions for the simple purpose of removing the appendix. I have no doubt whatever, from my own experience and from what I have seen of the work of my colleagues, that it is extremely dangerous to break down the barriers between an appendicular abscess and the rest of the peritoneal cavity." It is impossible in many instances, even with the greatest care, to avoid infecting the peritoneal cavity. Treves, too, is of much the same opinion, and says[4]: "As the surgeon, therefore, reaches what appears to be the starting point of the peritonitis, he must proceed with the utmost caution, and be not only prepared, but rather inclined to leave the actual *fons et origo mali* undemonstrated. . . . If the operator can rid the serous cavity of the effects of the perforation, he may very often leave the breach itself to be dealt with by natural means." Dr. White, in his paper,[5] quotes the opinion of some American surgeons to whom he submitted the question. Dr. McBurney said: "There are certainly some cases, not few in number, in which the appendix is so deeply embedded in the wall of the abscess, or so difficult to define at all, that to insist upon its discovery and complete removal would be to incur quite unjustifiable risk. . . . The accomplishment of this desirable result may, however, in some cases be accompanied by such great prolongation of the operation under unfavourable conditions, and by such great danger of seriously infecting fresh peritoneal surface, that one had better be content with properly evacuating, cleansing, and packing the cavity, leaving the appendix or its remnant to be disposed of by its obliteration in the wound-healing, or by its removal at a later and more favourable time through a second operation." Dr. Bull said that in cases operated upon after the seventh to the tenth day, or in "those where the

[1] *Ann. Surg.*, 1894, xix. 569.
[2] Quoted by White from *Boston M. & S. J.*, 1892, cxxvii. 105.
[3] *Am. J. M. Sc.*, 1894, cvii. 9. [4] *Brit. M. J.*, 1894, i. 519.
[5] *Loc. cit.*

abscess is distinctly circumscribed with firm walls and containing several ounces of pus, I have not attempted to remove the appendix. In many of those last mentioned it is found as a slough of the mucous membrane and in the pus. . . . The plan of always looking for the appendix is fraught with the risk of infecting the healthy peritoneum beyond the barrier of adhesions,—an immediate danger; the plan of leaving it under the circumstances mentioned exposes only a small proportion of patients to risk of subsequent attack or fistula, both remote dangers which can be met by secondary operations." Dr. Senn said: "It has been my habit for years in cases of acute appendicitis with extensive suppuration to simply incise, disinfect, and drain the abscess, unless the diseased appendix could be removed without any additional risk. I have seen a number of such cases recover permanently without any additional surgical interference. I regard persistent search for the appendix in such cases hazardous, as it often results in opening into the free peritoneal cavity and fatal septic peritonitis." Dr. Halsted said: "If there is only one abscess, and it can be evacuated without entering the uninvolved part of the peritoneal cavity, I almost invariably treat it simply by incision and drainage." Dr. Richardson said: "I have no doubt whatever that many cases which under conservative methods would recover are converted into cases of fatal general peritonitis by removing the appendix when it forms part of the wall of an abscess cavity. . . . I am fully convinced that many cases that are reported as cases of hopeless general infection are made hopeless and general by the rash methods frequently advocated to-day. In a localized abscess, especially if it has been going on a week or two, and the walls are firm, separation of adhesions, in my judgment and experience, is inadvisable,—more than inadvisable,—it is entirely unjustifiable. In a very large number of cases I can recall but two or three in which simple evacuation and drainage without removal of the appendix have not been followed by a cure thus far permanent. The ideal method of treating these cases is to tide the patient over the existing emergency by simple drainage, and, after permanent recovery has taken place, to remove the appendix." Such evidence is surely sufficient, and, as Dr. White says, it is "in accord with both clinical experience and surgical principles." The conclusions here set forth are practically the same as those expressed by Rutherford Morison,[1] Mitchell Banks,[2] Jordan Lloyd,[3] MacDougall,[4] and other surgeons who took part in the recent discussion on this subject at the meeting of the British Medical Association at Carlisle. It is obviously desirable to remove a diseased appendix when one can do so without increased danger to the patient; but to admit this is very different from suggesting that the appendix should be removed

[1] *Brit. M. J.*, 1896, ii. 1005. [2] *Ibid.*, 1007.
[3] *Ibid.*, 1005. [4] *Ibid.*, 1003.

in all cases. The fact is, as Dr. McBurney has pointed out,[1] that in cases of suppurative appendicitis we find we have to deal with two classes of abscess, which differ considerably in their management. In one kind we find the abscess has approached the parietes to such an extent that the abscess cavity can be opened—though frequently with some difficulty—without entering the general peritoneal cavity. All further manipulations are therefore made from the inside of the abscess cavity, the wall of which separates the infective pus from the general peritoneal cavity. Now, in such a case as this, where the appendix practically forms a portion of the abscess wall, an attempt to remove it would probably expose the patient to the risk of general peritoneal infection,—an inflammation of so fatal a character that, after what has been said, it would not be justifiable to expose the patient to it. It is true that by means of gauze we may often succeed in keeping the peritoneum free from infection in cases where the risk is considerable; but when we find that an appendix when not removed generally quiets down after the opening of the abscess, we must regard any interference with it, under the circumstances now referred to, as mischievous. In the other class of cases the abscess does not approach the parietes, but the abscess cavity and appendix are surrounded by agglutinated coils of intestine so that the whole mass lies more or less free in the peritoneal cavity. In operating on such cases we must necessarily open the general peritoneal cavity, and then by means of gauze and sponge packing we endeavour to isolate the affected coils of intestine from the general peritoneal cavity. We must then proceed to unravel the coils of gut until we come down on the imprisoned appendix and the surrounding abscess. In these cases the appendix can be, and we might say should be, removed; for it is generally under these circumstances easily isolated, and its isolation is a necessary part of the means taken for evacuating the abscess. The sponge packing enables us to keep the infected portions of intestine more or less under control; but the mortality in these cases is generally higher than in the former ones (provided that no attempt has been made to remove the appendix when it forms part of the abscess wall itself), on account of the anatomical relations of the abscess, which are necessarily more likely to allow of a general peritoneal infection.

The trite subject of the treatment of **buboes** following soft chancres is still one of interest both to the patient and the practitioner. These cases are of a most tedious character when treated by ordinary means, so that surgeons have been led to advise the excision of the affected glands whether suppurating or not. Such treatment, if one obtains union by first intention, undoubtedly shortens the ordinary duration of the disease; but

[1] *Ann. Surg.*, 1896, xxiii. 751.

I have been so impressed by the dangers of the operation, which involves an extensive dissection in the neighbourhood of the saphena and femoral veins, that it seems unjustifiable to adopt such radical measures in an uncomplicated case. We must reserve this procedure for such cases as have proved intractable to other means, or the slight mortality of buboes will be largely and unnecessarily increased. Dr. Clifford Perry [1] has recently had this subject under review, and considers that the best treatment of non-suppurating buboes of chancrous origin is by the intra-glandular injection of a one per cent. solution of benzoate of mercury—a method of treatment first advocated by Welander,[2] who claimed to have cured fifty-six out of seventy-eight cases without suppuration; and as the usual percentage of buboes which suppurate is about 42 per cent.[3] of the whole number, this result was extremely good. Dr. Taylor [4] recommended the injection of a solution of carbolic acid (twenty to forty minims of a solution of ten grains to the ounce), and said a cure was effected in eight to ten days, and Harvey [5] supported him; but this method, like that of the injection of bichloride of mercury, does not seem to have stood the test of time. If all that Perry [6] claims for the benzoate of mercury treatment be corroborated by further trials, we shall have a most useful addition to the treatment of these chancrous glands. He employs this remedy not only in the non-suppurating cases, but also in those in which suppuration is threatening, so long as fluctuation is not actually present. About twenty or thirty minims are injected at one or more points into the gland substance. This causes a burning pain for two hours, and generally some fever (99° F. to 104° F.) lasting for a few days. "In two or three days after the commencement of treatment the bubo is much diminished in size and resolution is taking place." A small quantity of pus generally forms in the centre; this is relieved by incision and the application of an antiseptic dressing. "In a few days all discharge ceases, the remaining induration rapidly subsides, and the bubo is cured." In twenty-two unselected cases treated by this method, he found the average time necessary to effect a cure to be about fourteen days. This is less than half the time usually occupied by cases that are excised; but is about the same time as that claimed by Woodward,[7] who advocates early incision and packing with iodoform gauze. As regards the treatment of suppurating buboes, Perry's conclusions are much the same as those of most of us. He finds simple incision, or incision and *curettement*, to be more or less slow; but, as already suggested, these means though slow are very safe. The best method of all, however, for these cases is one which is often

[1] *Am. J. M. Sc.*, 1896, cxii. 571.
[2] *Arch. f. Dermat. u. Syph.*, 1891, Heft 3, quoted by Perry.
[3] *International Encyclopædia of Surgery.* Edited by John Ashhurst, Jr., M.D., 1882, ii. 436.
[4] *Am. J. M. Sc.*, 1882, lxxxiii. 359. [5] *Med. News*, 1886, xlviii. 98. [6] *Loc. cit.*
[7] *Rep. Superv. Surg.-Gen. Mar. Hosp.* for 1892, p. 125.

applied to chronic abscesses, viz., the evacuation of the pus, followed by distension of the cavity with a ten per cent. mixture of iodoform in vaseline. This, though not new then, was advocated by Hayden in 1895.[1] Personally, I prefer a ten per cent. emulsion of iodoform in glycerine. One or two injections are generally sufficient, and the majority of cases get well in the course of two or three weeks. It is only when these milder means have failed that we should adopt *curettement*, or the more serious operation of excision.

JAMES SWAIN.

## Figures and Tables

**Figure f1:**